# Validation of the Norwegian version of the Parents’ Evaluation of Aural/ Oral Performance of Children (PEACH+) for children with typical hearing aged 12–72 months

**DOI:** 10.1371/journal.pone.0289898

**Published:** 2023-08-17

**Authors:** Lene Johansen, Thea Gray, Christiane Lingås Haukedal, Nina Jakhelln Laugen, Vasiliki Diamanti, Ulrika Löfkvist

**Affiliations:** 1 Department of Special Needs Education, Faculty of Educational Sciences, University of Oslo, Oslo, Norway; 2 Department of Psychology, Norwegian University of Science and Technology (NTNU), Trondheim, Norway; 3 Department of Public Health and Caring Sciences, Uppsala University, Uppsala, Sweden; Lamar University, UNITED STATES

## Abstract

**Background:**

The Parents’ Evaluation of Aural/Oral Performance of Children (PEACH+) is a parent reported questionnaire. It was first developed in Australia (2007) to assess the effectiveness of hearing devices in young children, and to register how oral children under the age of five hear and communicate with others.

**Objective:**

No validated version of the Norwegian translation of PEACH+ exists. This study therefore aims to evaluate the validity and reliability of a back-translated Norwegian version of the PEACH+, from a sample of Norwegian children with typical hearing.

**Methods:**

Parents of 255 children with typical hearing between 12 and 72 months were recruited through kindergartens and social media platforms. Participants were asked to fill in the PEACH+ questionnaire on behalf of their child, in a digital format.

**Results:**

High internal consistency (Cronbach’s Alpha = .917) and satisfactory item-total correlation were found (.342-.678).

**Conclusion:**

The Norwegian translation of PEACH+ shows good psychometric properties that are similar to the original version (Ching and Hill, 2007) and that of other translations. The PEACH+ questionnaire is therefore valid to use in a Norwegian context.

## Introduction

Validation of clinical instruments are crucial to obtain reliable and useful results that can be used to guide clinicians and caregivers. It is important that an instrument that is said to target certain areas, also measure what it claims to measure. To make sure that a translated instrument like a parent questionnaire can be used in clinical practice in other cultural and linguistic contexts than the country of origin, it is also necessary to conduct validation studies. For a parent questionnaire which measures different listening abilities, it is essential to initially perform a validation study in a cohort of typically hearing children. The current validation study of typically hearing children investigates if the Parents’ Evaluation of Aural/Oral Performance of Children (PEACH) [1] is valid and suitable to be used within a Norwegian context. If so, it will later primarily be used for young children with hearing loss.

The introduction of universal newborn hearing screening and innovations within hearing technology has enabled the possibility of early diagnosis and habilitation of newborns with hearing loss [1]. Early access to sound through amplification is essential for auditory stimulation, and thus for the spoken language development of children who are hard of hearing [2–5].

Outcome evaluation of hearing aids in all age groups is a key component of the hearing aid fitting process [6]. However, this can be challenging in the paediatric population, particularly in the prelingual phase when children cannot provide caregivers and audiologists with oral feedback on how they hear with their hearing aids. Objective verification of hearing amplification can be performed in hearing clinics by measuring *cortical auditory potentials* (CAEPs) evoked by speech at conversational level [7]. However, objective measures fall short when it comes to providing information about how the amplification works in the everyday lives of children. Furthermore, objective measures do not yield information about the development of aural and oral performance. Therefore, subjective tools are needed for a more dynamic and multi-tooled approach to clinically monitor auditory development and the effectiveness of amplification as the child continues to grow and develop.

Parent-reported questionnaires are cost-effective and frequently used for assessments of children’s functioning and are considered to be a more representative source of information than questionnaires carried out in structured and clinical settings by professionals who often do not know the child [8]. The PEACH questionnaire evaluates the effectiveness of amplification in infants and children with hearing loss through systematic use of caregiver observations [1]. Parents report their child’s hearing, listening and verbal communication, both in quiet and in noise. The questionnaire is norm-referenced, and based on the results of Australian children with typical hearing aged 0–5 years [1]. In the most recent version of the instrument called PEACH+ [9], an additional scale is included in which caregivers are asked how easy or difficult they believe it is for their child to demonstrate the given behavioural pattern in a range of different situations.

The original PEACH instrument yielded good reliability (Cronbach’s Alpha 0.88) and a meaningful factor structure [1]. Initially, the PEACH was administered as an interview with the caregiver after a dedicated observation period (PEACH Diary). However, a later validation study of the PEACH administered as a questionnaire (PEACH Rating Scale) revealed psychometric properties and age norms very close to the original study [6]. The PEACH has been translated and adapted to various cultural and linguistic contexts, including Malay [8] Spanish [10], Swedish [11] and Chinese [12]. Overall, the psychometric properties of the PEACH are confirmed across these diverse contexts, however the “ease of listening” scale that was added in the PEACH+ version [9] has so far only been subject to one validation examination in Malay [13]. Furthermore, a Finnish translated version of PEACH+ is available [14].

Among the auditory inventories and questionnaires that are internationally recognized, only a few of them have been translated and adapted into Norwegian. The LittlEARS^®^ Auditory Questionnaire (LEAQ) is among them [15, 16], as well as a previous translation of PEACH, however the latter has not been validated for use in Norwegian. Adaption of a questionnaire that is based on a specific language and culture will not, and should not, be constrained to translation alone. An empirical study is necessary to establish the validity and reliability of the translated and adapted questionnaire [9]. A validated screening tool is also essential in evidence-based practice [17], and is much needed in the follow-up evaluation of Norwegian children with hearing loss. Therefore, the objective of this study was to investigate psychometric properties of the PEACH+ in a Norwegian population, including both the original PEACH score and the ease of listening score, and to provide age norms for children aged 1–6 years with typical hearing who use oral communication.

## Materials and methods

The current study is part of a larger project,” Bedre verktøy” (Better Tools). The study is a collaboration between Norwegian University of Science and Technology (NTNU), Trondheim, and the Department of Special Needs Education, University of Oslo, Oslo. The Regional Committee for Medical and Health Research Ethics evaluated the study and waived the need for an ethical approval (No. 140084), because the data collection procedure provided anonymity of the participants throughout the process.

### Recruitment and inclusion criteria

The recruitment process took place mainly through preschools. Potential participants were contacted by test administrators and asked to distribute information about the study to parents in their preschool units, including a link to an informed consent and the online questionnaire. The same information was also shared in social media, such as parent groups on Facebook. The recruitment procedure did not allow us to assess the total number of parents who were invited to the study; thus, the response rate is unknown. Participants were recruited from nine of Norway’s 11 counties, including both urban and rural areas. They were not asked if they lived in urban or rural areas, and their participation was anonymous. Therefore, the proportion of urban vs. rural participation is unknown.

Participants were selected according to the following inclusion criteria: the child had to (1) be 12–72 months, (2) have typical hearing, and (3) have been well for the last two weeks prior to the assessment (i.e. not have had any airway infections or cold virus that could have impacted their hearing at the time of assessment). The total number of respondents comprised 292 caregivers, who each responded for one child. Thirty-seven participants were excluded due to the presence of hearing loss (n = 7), incomplete questionnaires (n = 10), being outside the age criteria (n = 3) or for having been sick within the previous two weeks (n = 17).

### Participants

**Fig 1:**
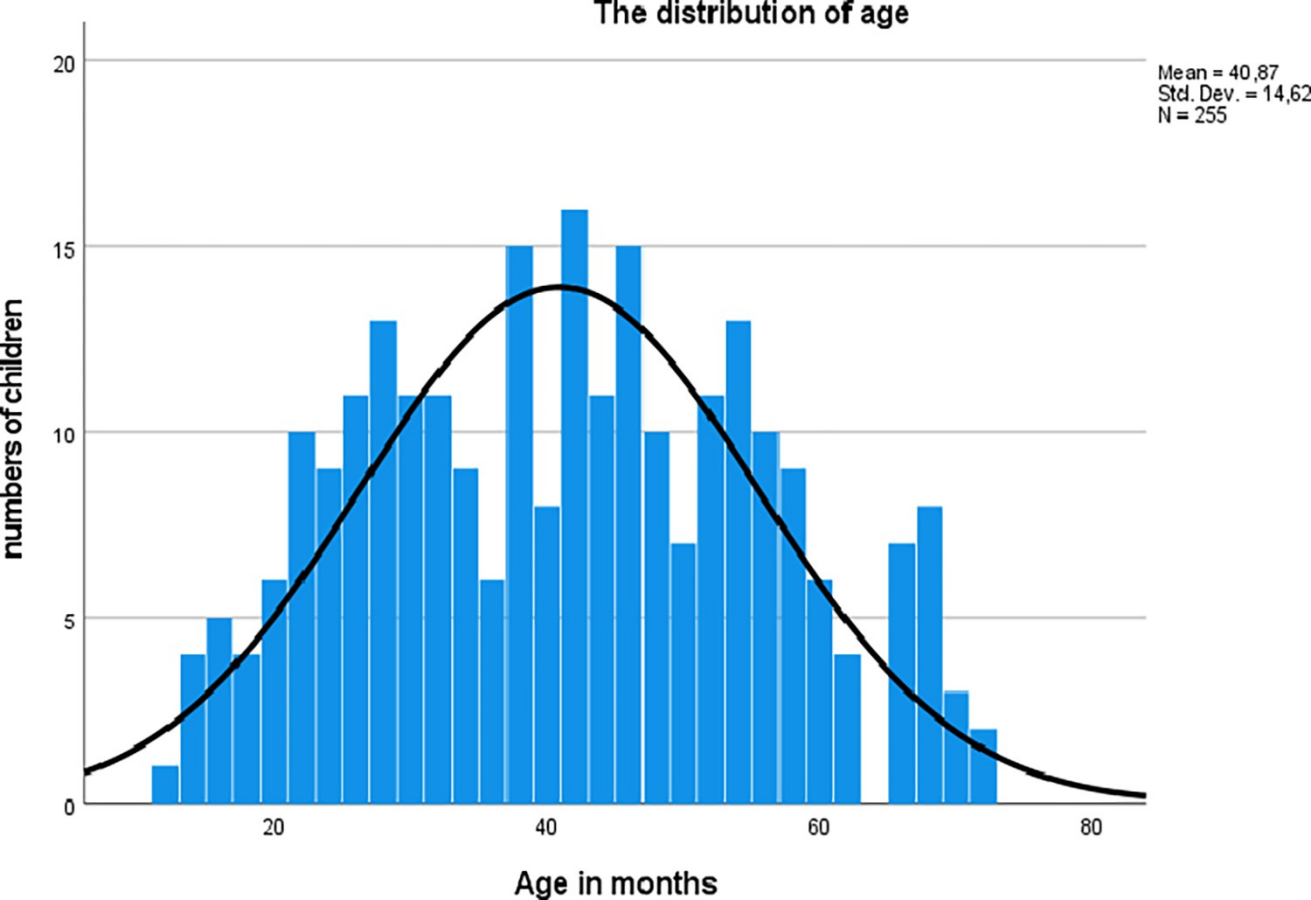
The distribution of age in children with typical hearing (12–72 months).

The final sample included caregivers of 255 children with typical hearing, where 124 of the children were of female sex (49%) and 131 were of male sex (51%). The children’s ages ranged from 12 to 72 months (Mean: 40.87, SD: 14.62). The age distribution is shown in Fig 1.

Eighty-two percent of the participating children (n = 217) had passed neonatal hearing screening, while 18% (n = 46) did not report or were not sure if their child had passed or not. Most of the children, 99% (n = 252), attended preschool. The majority of the respondents were mothers (82%). The level of education was relatively high, as 31% reported having a bachelor degree and 52% had a master degree or PhD.

#### Measures

*PEACH+*. The PEACH+ consists of 12 items and two background questions. All items describe listening behaviors. The parent reports how often the behavior occurs (the PEACH scale), and how easy or difficult they believe the situation is for the child (the ease of listening scale). Responses are provided on 5-point Likert scales, ranging from 0 = *never* to 4 = *always* on the PEACH scale, and from 0 = *very difficult* to 4 = *very easy* on the ease of listening scale.

Items 3, 5, 7, 9 and 11 represent quiet situations, while items 4, 6, 8, 10 and 12 represent situations in noise. Per the creators’ instructions of the original PEACH measure, the questionnaire was scored by summing the item scores, dividing by 20, before multiplying by 100 to calculate the percentage. The total score for both scales was derived by adding the sum for quiet (a) and noise (b) and then divided by 2.

*Translation of PEACH+ into Norwegian*. PEACH+ was originally developed in Australia for use in English-speaking children. The already existing PEACH version in Norwegian had not been translated according to best practice [18]. Therefore, a new adaptation of the PEACH+ into Norwegian was adapted [19] with the ultimate goal of standardizing the questionnaire into Norwegian. Both the instructions and the questionnaire of the PEACH+, underwent a new «back to back translation». This entails that the questionnaire was first translated into Norwegian, before it was translated back to the original language, to ensure that the Norwegian version does not deviate too much from the original [18]. The translation was then discussed by an expert panel consisting of professionals from different clinical backgrounds such as ear nose throat doctors, educational audiologists, psychologists, and audiologists. Based upon the discussions, the translation was adjusted before joint agreement. In the final version, the instructions are slightly modified due to the Norwegian version being distributed directly to caregivers, whereas the instructions in the original PEACH+ refers to the audiologist or other professional who presents the questionnaire to the caregiver. After the PEACH+ had been reviewed, it was approved by Theresa Ching, the developer of PEACH+ [20]. The Norwegian PEACH+ questionnaire is available on the FigShare website [19], and the data from the current study is published in NTNU Open Research Data [21].

*Inclusivity in global research*. Additional information regarding the ethical, cultural, and scientific considerations specific to inclusivity in global research is included in the Supporting Information (S1 Questionnaire).

*Statistical analysis*. Means, median, standard deviation (SD) and ranges were calculated for all variables. The first item of PEACH+ concerns use of amplification and was therefore removed as it was not relevant in our sample of children with typical hearing. The first item in PEACH+ is «How often has your child worn his/her hearing aids and/or cochlear implant?» and was not included as the participating children in this study all have typical hearing. The second item was also related to listening experiences with hearing technology and therefore removed, in line with the original validation [1], which reported low inter-item correlations for this item. The same item was also removed in the Spanish and Swedish validations [10, 11]. To check if the PEACH data was normally distributed a visual inspection was performed using histogram and Q-Q.

The internal consistency was examined by calculating the Cronbach’s Alpha coefficient for both total scores and the subscales [22]. Corrected item total correlations were calculated to explore each item’s contribution to the overall scores and overall internal consistency. Associations between age and total scores were examined with bivariate correlations and were generated as scatterplots.

Principal component analyses (PCA) were performed to examine the structure of relationships between the items. In order to extract the factors, we applied PCA with varimax rotation, and only factors with an eigenvalue larger than one were included. For all analyses, we applied an alpha of 0.05.

## Results

Table 2 shows means, standard deviation (SD) and ranges for the subscales Quiet and Noise, as well as the Total score, for each age group participating in the study. Visual inspection of histograms and Q-Q plots revealed normal distributions (Table 1).

**Table 1:** Descriptive Norwegian PEACH+ results for all study participants and by age group (N = 255).

		PEACH scale			Ease of Listening scale		
Age (months)	N	Quiet Mean (SD)	Noise Mean (SD)	Total Mean (SD)	Quiet Mean (SD)	Noise Mean (SD)	Total Mean (SD)
12–23	35	79.0 (13.7)	71.4 (13.0)	75.2 (12.6)	77.7 (15.1)	68.0 (18.8)	72.9 (16.0)
24–35	60	84.3 (13.2)	76.0 (14.1)	80.2 (12.5)	81.9 (12.8)	71.9 (17.1)	76.9 (14.2)
36–47	73	86.2 (10.2)	76.7 (12.4)	81.5 (10.5)	84.0 (11.3)	73.9 (14.2)	78.9 (11.7)
48–59	57	88.5 (9.3)	78.3 (11.9)	83.4 (9.9)	84.1 (13.5)	73.7 (16.3)	78.9 (14.3)
60–72	30	87.7 (11.5)	77.8 (13.0)	82.8 (11.5)	85.2 (10.5)	76.5 (11.7)	80.8 (10.0)
Total	255	85.5 (11.7)	76.3 (12.9)	80.9 (11.5)	82.8 (12.7)	72.9 (15.9)	77.8 (13.4)

*Note*: SD = standard deviation.

### Internal consistency

Cronbach’s Alpha (α) was calculated for the PEACH scale and the Ease of listening scale, as well as the noise and quiet subscales. For the PEACH scale, the total score yielded high internal consistency (α = .83) and satisfactory levels for the noise and quiet subscales (α = .70 and .71, respectively). Regarding the Ease of Listening score, levels were α = .88 for the total score, α = .75 for the quiet subscale and α = .81 for the noise subscale.

The correlation between each item and the composite score to which it belongs, i.e., corrected item-total correlation, are presented in Table 2. Correlations ranged from .277 to .670. Cronbach’s Alpha was re-calculated by removing each item in turn, in order to find out if each item contributes to the overall internal consistency. The only item that made an impact, when removed, was item 12, which resulted in a slight Cronbach’s Alpha increase of the PEACH score.

**Table 2:** Corrected item-total correlations and Cronbach’s Alpha if item deleted.

		PEACH score		Ease of listening score	
Item	Description	corr_i-t_	α if deleted	corr_i-t_	α if deleted
3	Respond to name in quiet	.584	.81	.589	.86
4	Respond to name in noise	.617	.81	.643	.86
5	Follow verbal instructions in quiet	.544	.82	.514	.86
6	Follow verbal instructions in noise	.647	.81	.670	.86
7	Participate in conversation in quiet	.567	.81	.573	.87
8	Participate in conversation in noise	.587	.81	.657	.86
9	Follow story read aloud	.430	.83	.458	.87
10	Understand speech during transport	.477	.82	.584	.87
11	Recognize voices of familiar persons	.508	.82	.422	.86
12	Recognize sounds in environment	.277	.84	.587	.87

*Note*: corr_i-t_ = corrected item-total correlation. α if deleted = Cronbach’s α if this item is deleted from the total scale.

### Factor analysis

To detect structures and commonalities in the relationships between the items in the PEACH score, we conducted a principal component analysis (PCA) with varimax rotation [20]. The Kaiser Meyer-Olkin and Bartlett tests of sphericity suggested that the data were acceptable for factor analysis. With an eigenvalue cut-off of 1.0, the unrotated PCA split the items into two factors. After eliminating factor loadings below .3, a varimax rotation was performed, resulting in factor loadings ranging from .363-.842. The factors accounted in total for 51.9% of the variance, suggesting a moderate degree of total explained variance.

To test the constructs of the subscales Quiet and Noise, two additional PCAs with varimax rotation and Kaiser normalization were carried out. The results are shown in Table 3 as PCA 2. For the subscale Quiet, only one factor had an eigenvalue over 1.0 and explained 46.7% of the total variance. Similarly, for subscale Noise, one factor had an eigenvalue over 1.0 and accounted for 46.7% of the total variance.

**Table 3:** Factor analysis PCA for the Norwegian PEACH+.

	PEACH score				Ease of Listening score			
	PCA 1		PCA 2		PCA 3		PCA 4	
Item	Factor 1	Factor 2	Quiet	Noise	Factor 1	Factor 2	Quiet	Noise
3	.842		.691		.734		.704	
4	.880			.767	.842			.784
5	.545	.387	.707		.673	.325	.742	
6	.665	.393		.804	.812			.813
7	.375	.583	.722		.354	.590	.737	
8	.440	.542		.736	.573	.473		.767
9		.746	.631			.785	.653	
10		.707		.614		.730		.688
11		.606	.662		.324	.665	.710	
12		.363		.430	.325	.644		.709
Total	26.7%	25.2%	46.7%	46.7%	30.9%	28.0%	50.4%	56.8%
Corr	.87		.74		.91		.77	

*Note*: PCA = principal component analysis. Total = Proportion of total variance explained. Corr = correlation between factors.

The same set of PCA was conducted for the Ease of Listening score (PCA 3 and 4 in Table 3). PCA 3 yielded two factors, quite similar to the factors in PCA 1, with a total explained variance of 58.9%. Each of the PCAs for the Quiet and Noise subscales resulted in one factor for each of the subscales, with moderate to high loadings. For both the PEACH and Ease of Listening scores, the factors were highly correlated. In addition, the PEACH and the Ease of Listening scores were correlated (r = .79, *p* < .001).

### The PEACH+ total score and chronological age

**Fig 2:**
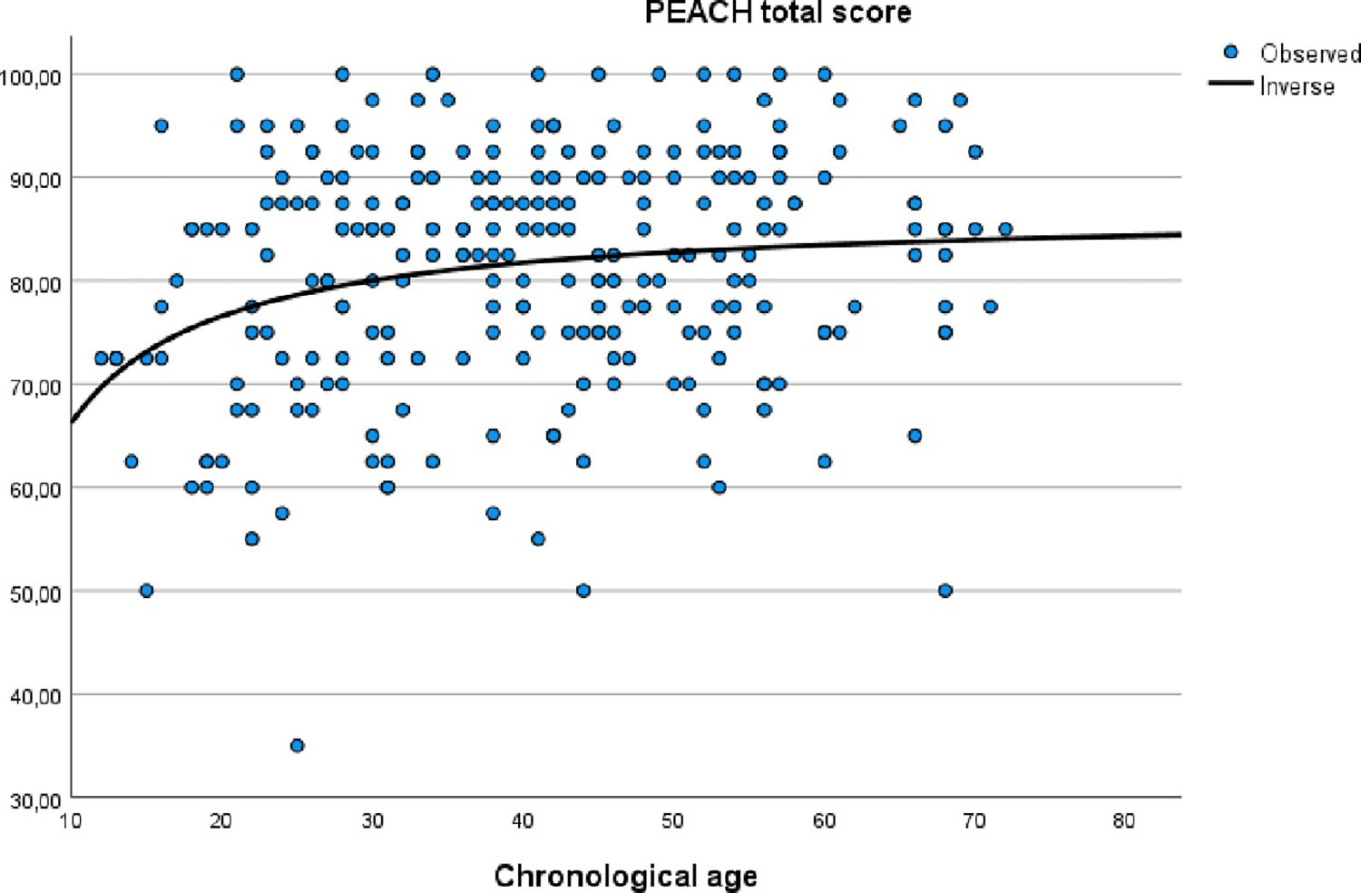
Relationship between PEACH total score and chronological age in children with typical hearing.

**Fig 3:**
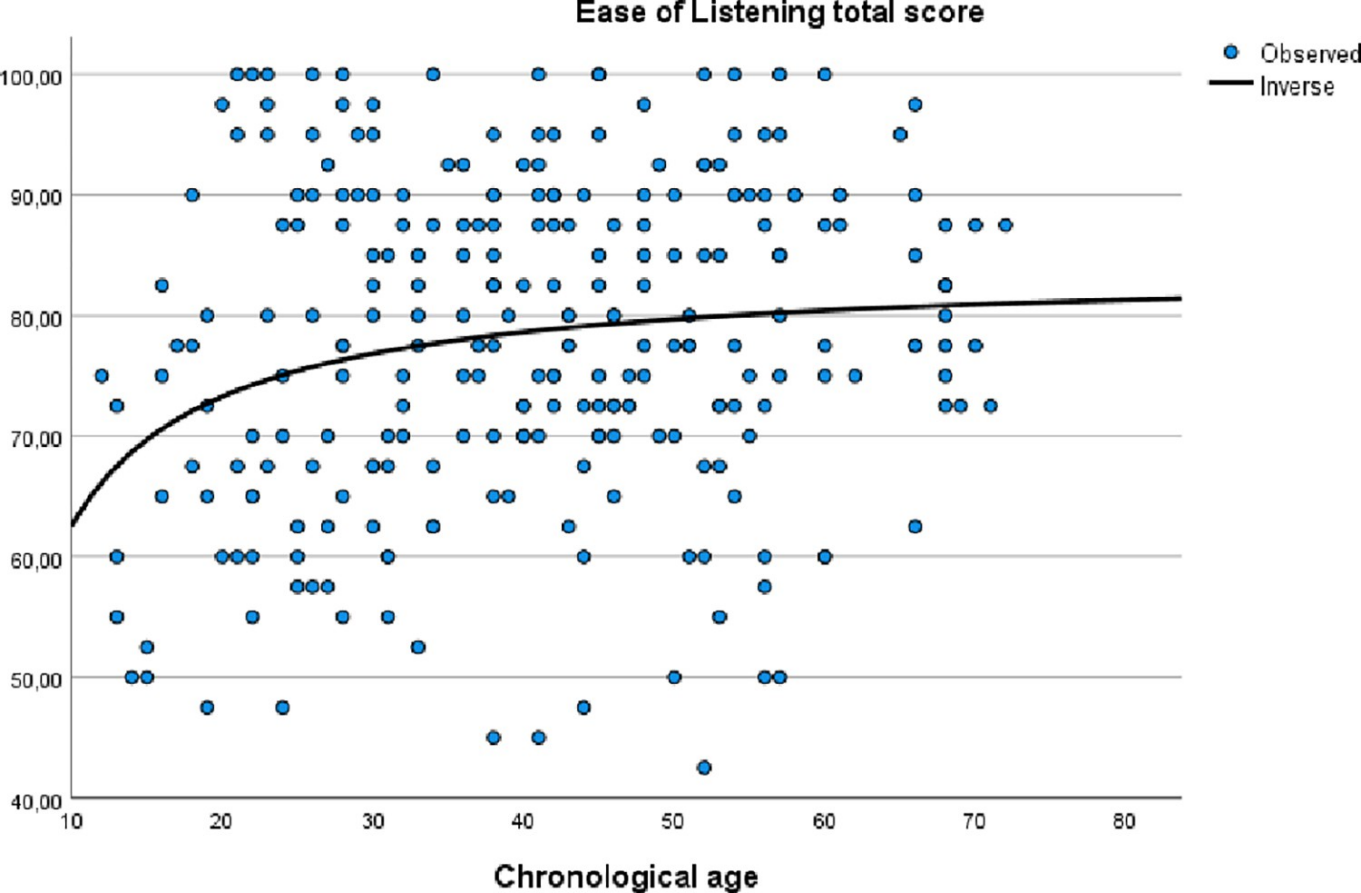
Relationship between Ease of Listening total score and chronological age in children with typical hearing.

The relationship between the PEACH total score and age was explored by an inverse regression model, as in the original version [1]. The same was performed with the Ease of listening score. The results are shown as scatterplots in Figs 2 and 3.

A visual examination of the inverse regression curves shows that the scores increase steadily from 10 months until 40 months. After that, the curves flatten out. Visually, the correlations between the total scores and age look divergent, with large deviations from the regression line. Correlation analyses show that there are weak, but significant correlations between age and the total scores (PEACH: r = .19, *p* = .002; Ease of Listening: r = .16, *p* = .01).

## Discussion

The purpose of this study was to examine the psychometric properties of the Norwegian translation of the PEACH+ in a sample of children aged 12 to 72 months with typical hearing. Results show that the Norwegian translation of the PEACH+ has high internal consistency, which is similar to the internal consistency of the original Australian/ English version [1], as well as the Swedish [11], and Spanish version [10]. Although the corrected item-total correlations were not as strong as those found in the Australian/ English, Swedish or Spanish validations, the Norwegian version of the PEACH+ shows a moderate corrected-item total correlation. One item, 12a, had a lower corrected item-total correlation, and removing the item increases the overall Cronbach’s Alpha. However, as the increase is only negligible the item was kept in the scale.

As with the original Australian English version, the first PCA showed that the items of the PEACH score were split into two different factors. However, the factor structure was not comparable to the structure found by Ching and Hill [1]. Rather, the structure is more similar to that reported in a Swedish sample [11]. Possibly this could be due to the fact that Ching and Hill [1] included both children with typical and reduced hearing in their analysis, whereas Brännström et al. [11] included only typically hearing children, as in the present study.

The factor analyses for the quiet and noise subscales showed that each subscale had only one factor with an eigenvalue over 1.0. This is similar to the findings in the original version [1] and the Swedish validation of PEACH [11]. Also similar to Ching and Hill [1] the current study obtained reasonably high factor loadings (>.6). As in the Swedish study [11], we found high correlations between the quiet and noise subscales. This could mean that they are not separate scales, but rather measuring the same construct. If that is the case, the use of the total PEACH score would be preferred over the use of the noise and quiet subscales. However, as pointed out by Brännström and colleagues [11], it is possible that the differentiation between quiet and noisy situations are more meaningful for children with reduced hearing than for children with typical hearing. The factor structure therefore needs to be re-investigated in populations with various degrees of hearing loss in future studies.

Regarding the Ease of Listening score, this is a relatively new addition to the PEACH instruments, and its psychometric properties has not—to our knowledge—been previously reported except for in Malay showing good internal consistency [13]. Our results also suggest good internal consistency, and yielded factor structures and loadings similar to the PEACH scale. In addition, for the Ease of Listening score, high correlations were found; moreover, the PEACH total score and the Ease of Listening total score were also highly correlated. If these results are replicated in future studies in DHH populations, the addition of the Ease of Listening scale may not be an important addition of the instrument. That said, future studies might also find that the distinction between listening performance and ease of listening is more useful in DHH populations than in typically hearing populations.

Previous studies show in general a strong relationship between age and the PEACH total score, typically with a rapid increase the first 20 months and then gradually reaching a ceiling effect around 40 months; high scores (>80%) after 40 months [1, 8, 10, 11]. In the current study, the relationship between the PEACH total score and age shows the same general pattern, however, as we did not include children under the age of 12 months, less of the steep curve is evident. Although the correlation between age and total score is still significant, it is rather weak. For more complete date to be used as norms for Norwegian children, an additional study including younger children (<12 months of age) is recommended. The flattening of the curve after the age of 40 months also suggests that the PEACH+ is primarily clinically useful for children below this age, and less so for older children.

## Limitations

The Norwegian validation of the PEACH+ questionnaire has some potential limitations. First, a test-retest was not conducted. If a test-retest yielded similar results, this would have further strengthened the reliability of the Norwegian PEACH+. Second, there was a smaller size of the group of participants between 12–23 months in relation to the other age groups. Third, the current study did not have information about non-participants i.e., subjects who received information about the study and who choose not to take part, this could have had an effect on how generalizable the results really are. Fourth, restricted variability in caregivers’ socioeconomic status (SES), in this case educational level. Thus, further research should aim to investigate auditory behaviour in children of families with low SES. In this study 83,6% (n = 213) of the caregivers (caregiver 1, see Table 2) had high educational level, however this did not seem to have a significant impact on the PEACH+ total score.

Fifth, it might be considered a weakness that there was no objective measure of the children’s hearing prior to partaking in the study, which means that this study is reliant on the caregiver being aware of their child’s hearing status. Other validations [1, 8, 10] have conducted objective measures of hearing i.e. passed OAE-screening as part of their inclusion criteria, while the original PEACH [1] had as an inclusion criteria that the child (in which the caregiver was answering on behalf of) must have passed the newborn hearing test (OAE). Objective hearing tests are likely to strengthen overall validity as there is a higher assurance that the participants in the sample have typical hearing and therefore a higher confidence that they have measured what they intended to measure i.e. listening development in children with typical hearing. One more limitation was that we were unable to compare the PEACH results with another similar instrument. There is currently no other standardized parent questionnaire that target listening behaviour of young children in Norway.

Finally, another limitation was related to the mode of data collection. The administration of the Norwegian version was done digitally and therefore this study relied on the caregivers’ knowledge, and awareness of their children’s hearing status. There were 17,7% (n = 45) of the parents who reported that they did not know whether or not their child passed neonatal hearing screening. Although the parents did not know, we chose to include these participants in our sample. This decision was made on the basis that if the child did not pass the neonatal hearing screening it would have prompted a response from the Norwegian health care system (according to clinical routine), and it is therefore reasonable to assume that the parents would have been notified and therefore aware of the result [23].

## Conclusion

The Norwegian PEACH+ has psychometric properties similar to that of the original PEACH version [1] and that of other validated translations of PEACH. We therefore consider it a useful instrument for clinical practice to evaluate the effectiveness of hearing devices in children between 12–72 months and to monitor auditory development in the same age group. However, the total score may be more useful than the subscales, and the Ease of Listening scale may not provide any additional information, unless future studies provide stronger evidence for its factor structure. This study was based on children with typical hearing, further research should be conducted to investigate the findings in children with hearing loss, but also in cohorts with typical hearing from families with more diverse SES background.

## Supporting information

S1 Questionnaire Inclusivity in global research. (DOCX)
